# Research on the Working Performance and the Corresponding Mechanical Strength of Polyaluminum Sulfate Early Strength Alkali-Free Liquid Accelerator Matrix Cement

**DOI:** 10.3390/ma15228086

**Published:** 2022-11-15

**Authors:** Lin Wang, Xinxin He, Chunxue Shu, Zicheng Wei, Hui Wang

**Affiliations:** 1 School of Civil and Transportation Engineering, Beijing University of Civil Engineering and Architecture, Beijing 100044, China; 2 School of Civil and Environmental Engineering, Ningbo University, Ningbo 315000, China

**Keywords:** accelerator, setting time, mechanical properties, hydration heat, hydration products

## Abstract

Liquid accelerating agents have the advantages of simple operation and fast construction, and have become indispensable admixtures in shotcrete. However, most liquid accelerating agents in the market at present contain alkali or fluorine, which adversely affect concrete and seriously threaten the physical and mental health of workers. Therefore, in view of the above deficiencies, it is necessary to develop a new type of alkali-free fluorine-free liquid accelerating agent. In this paper, the polyaluminum sulfate early strength alkali-free liquid accelerator is prepared using polymeric aluminum sulfate, diethanolamine, magnesium sulfate heptahydrate and nano-silica. The influence of this agent on the setting time of fresh cement paste and compressive strength of the corresponding cement mortar is determined. Thermogravimetric analysis curves, X-ray diffraction and scanning electron microscopy images are obtained to investigate the mechanism. Findings show that the initial setting time and the final setting time of cement paste are 2 min 30 s and 7 min 25 s. The compressive strengths of cement mortar cured for 1 d, 28 d and 90 d are 2.4 MPa, 52.2 MPa and 54.3 MPa respectively. Additionally, the corresponding flexural strengths are 3.4 MPa, 9.8 MPa, 11.8 MPa. When the mass rate of accelerator is 7%, the mechanical strengths of cement mortar are the highest. The additions of fly ash and blast furnace slag can affect the mechanical of cement mortar mixed with accelerator. When the mass ratio of the fly ash and blast furnace slag is 15%, the mechanical strengths of cement mortar reach the highest. Moreover, the hydration heat release rate of cement is increased by the accelerator and the corresponding time of hydration heat peak is decreased by the accelerator. The accelerator can decrease the amount of needle-like hydration products and improve the compactness. The mechanical strengths are improved by consuming a large amount of Ca(OH)_2_ and forming more compact hydration products. It is recommended that the optimum dosage range of the polyaluminum sulfate early strength alkali-free liquid accelerator is 7%.

## 1. Introduction

A quick-setting agent is an admixture that can quickly condense and harden concrete, which is widely used in tunnels, mines and other projects [1,2,3,4]. In recent years, with the development of the spray concrete wet spraying process [5,6], liquid quick-setting agent has become an indispensable material in the construction of sprayed concrete, which has the characteristics of significantly reducing the amount of rebound and dust concentration in the spraying construction process. Moreover, the thickness of single injection is increased and the speed of concrete condensation and hardening accelerated, gradually replacing the past powder quick-setting agent [7,8,9] From the perspective of green environmental protection, energy conservation and emission reduction, it is imperative to use large-scale wet spraying for the construction of sprayed concrete, and the demand for alkali-free liquid fast-setting agent has shown explosive growth [10,11,12,13]. Alkali-free liquid quick-setting agent has the advantages of green, environmental protection and high efficiency, which can not only ensure green environmental protection in the production process but also ensure efficient and rapid construction during the construction process [14,15]. Therefore, the characteristics of green, environmental protection and high efficiency will surely be the mainstream trend of the development of alkali-free liquid fast-setting agents in the future [16]. At present, whether it is the needs of society or the needs of national development strategy, the development trend of alkali-free liquid quick-setting agents have inevitably taken into account both technical properties and green environmental protection, especially in recent years [17,18].

The alkali-free liquid quick-setting agents began to be researched in European and American countries in the 1990s [19,20,21]. The main research idea of alkali-free liquid quick-setting agent is to find new pro-coagulation materials to replace the alkali metal salts in liquid quick-setting agents. Researchers have carried out a lot of efforts on liquid quick-setting agents prepared with aluminum sulfate as the main procoagulant component [22,23]. Quick-setting agents with excellent application performance and stability have been developed by BASF and Sika [24,25]. Most researchers select the aluminum sulfate as the main coagulation component of liquid quick-setting agents; however, aluminum sulfate shows low solubility and easy hydrolysis [26,27].

The alkali-free liquid quick-setting agents with aluminum sulfate used as the main fast-setting component have attracted some scholars’ attention [28,29]. Now, aluminum sulfate is mostly used as the main pro-coagulation component at home and abroad. However, Al^3+^ is difficult to dissolve in water. The aluminum ions are prone to hydrolysis reaction and flocculent precipitation. With the extension of storage time, crystallization and precipitation occur easily [30,31]. At present, most of the research on liquid accelerators has had problems, such as substandard strength and poor adaptability. In this study, a new liquid accelerator with high storage stability and the improving effect on the mechanical strength has been investigated. The inner mechanism of the liquid accelerator on the mechanical strength of cement mortar has been revealed.

We aimed to prepare a polyaluminum sulfate early strength alkali-free liquid accelerator by mixing the polymeric aluminum sulfate, diethanolamine, magnesium sulfate heptahydrate and nano-silica. The setting time of the fresh cement paste with this accelerator is investigated. The compressive and flexural strengths of the corresponding cement mortar are tested. The mechanism of the agent on the macro performance of cement-based materials is investigated by the thermogravimetric analysis curves, X-ray diffraction and scanning electron microscopy (SEM).

## 2. Experimental

### 2.1. Raw Materials

Polyaluminum sulfate, diethanolamine, Epsom salt and ethylene glycol produced by Shanghai MacLean Biochemical Technology Co., Ltd., Shanghai, China were used for manufacturing the polyaluminum sulfate early strength alkali-free liquid accelerator. The average particle size of nano-silica produced by Shanghai MacLean Biochemical Technology Co., Ltd., Shanghai, China is 30 ± 5 nm. QM168C stabilizer is produced by Dongguan Jianxing New Material Technology Co., Ltd., Dongguan, China, and 0.1 mol/L dilute sulfuric acid is produced by Shanghai Darui Fine Chemicals Co., Ltd., Shanghai, China. The deionized water produced by Shanghai Chuangsai Technology Co., Ltd., Shanghai, China with a density of 1.000 g/mL is selected.

In this experiment, P·I 42.5 produced by Fushun Cement Co., Ltd., Fushun, China is used as the reference cement. Ordinary Portland cement including P·O 42.5 Nanfang cement, P·O 42.5 Jinyu cement and P·O 42.5 Fushun cement are produced by South Cement Co., Ltd., Hanzhou, China, Jinyu Cement Co., Ltd., Zaozhuang, China and Fushun Cement Co., Ltd., Fushun, China, respectively.

Secondary fly ash with the specific surface area of 386 m^2^/kg produced by Beijing Jingyeda New Building Materials Co., Ltd., Beijing, China and S95 blast furnace slag powder (BFS) (specific surface area of 452 m^2^/kg) produced by Jintaicheng Technology Group Co., Ltd., Shahe, China are used as mineral admixture. The chemical compositions of different type of cement are shown in Table 1.

The water-reducing agent is polycarboxylate superplasticizer (water reduction of 25%) produced by Jiangsu Subote New Materials Co., Ltd., Nanjing, China and naphthalene superplasticizer (water reduction of 20%) produced by Hebei Guangming Chemical Technology Co., Ltd., Shijiazhuang, China. The experimental water is deionized water produced by Shanghai Chuangsai Technology Co., Ltd., Shanghai, China. The density of deionized water was 1.000 g/mL.

### 2.2. Sample Preparation

**Figure 1:**
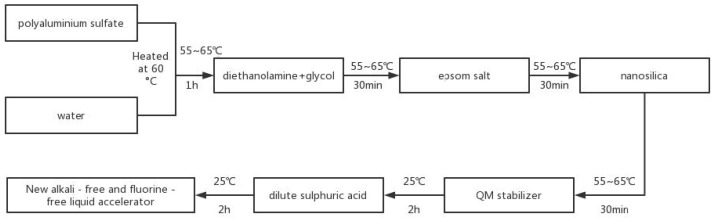
Process of the preparing of new alkali-free and fluorine-free liquid accelerator.

The preparing method of the new alkali-free and fluorine-free liquid accelerator is shown in Figure 1. Each component is weighed according to the mass percentage, and the proportions. The mass ratio of polyaluminum sulfate: diethanolamine: ethylene glycol: magnesium sulfate heptahydrate: nano-silica: QM stabilizer: dilute sulfuric acid: water is 57:6:1:2:2:3:1:28. The new alkali-free and fluorine-free liquid accelerator is made by the following steps.

The fresh cement paste can be produced by the following steps. NJ-160 cement paste mixer produced by Wuxi Jianyi Instrument Machinery Co., Ltd., Wuxi, China is used to stir the cement paste. The stirring process can be divided into the following steps. Firstly, the weighed gelling material and water are added into the stirring pot then 30 s stirring with the speed of 62 r/min. When the stirring is finished, the liquid accelerator is injected with a 50 mL syringe to the cement paste mixer and stirred for 1 min. After the mixing is completed, the fresh cement paste is used for the measurement of the setting time by following the process in Chinese standard GB/T 35159-2017 [32]. JJ-5 cement mortar mixer produced by Wuxi Jianyi Instrument Machinery Co., Ltd., Wuxi, China is used for stirring the cement mortar. Firstly, the weighed cementitious material is put into the mixer and mixed at a speed of 140 r/min for 1 min, then the uniform mixed water and accelerator is added and stirred at a speed of 140 r/min for another 1 min. Finally, the standard sand is added and 2 min stirring with the speed of 285 r/min is carried out. When the stirring process is finished, the fresh mixture is poured into molds sized 40 × 40 × 160 mm^3^. The mixing proportion of specimens is shown in Table 2.

### 2.3. Measurement Methods

#### 2.3.1. Mechanical Strengths

The compressive strength of cement mortar is tested by using the YAW-300 microcomputer controlled electrohydraulic servo pressure testing machine produced by Shanghai Sansi Hengheng Machinery Manufacturing Co., Ltd., Shanghai, China. Three samples are required for the flexural experiment and six samples are required for the compressive test. The loading rates of the flexural and compressive strengths are 0.01 kN/s and 2.4 kN/s respectively. The measuring process is carried out referring to the Chinese standard GB/T 17671-1999 [33].

#### 2.3.2. Measurement of Hydration Heat

In order to test the hydration heat, the liquid accelerator with different dosage is mixed with water to prepare a mixed solution, then weighed cement is put into the sample bottle of isothermal calorimeter to mix evenly and is used for testing. The measuring process is carried out following the Chinese standard GB/T 12959-2008 [34].

#### 2.3.3. Measurement of SEM and XRD

The hardened cement paste with smooth surface at different curing ages is immersed in the absolute ethanol to prevent hydration. The samples are dried in a 60 °C drying oven for 3 days until the mass is constant. When these steps are finished, the samples of soybean size are sprayed with gold and moved for measurement with SEM. SEM model geminisem300 (Zeiss), producing area for Oberkochen, Germany. Some dried samples are ground and sieved through a 0.075 mm square-hole sieve. The measuring process is implemented according to the Chinese standard SY/T 5162-1997 [35].

## 3. Experimental Results and Analysis

### 3.1. Basic Properties of Cement Paste with Accelerator

**Figure 2:**
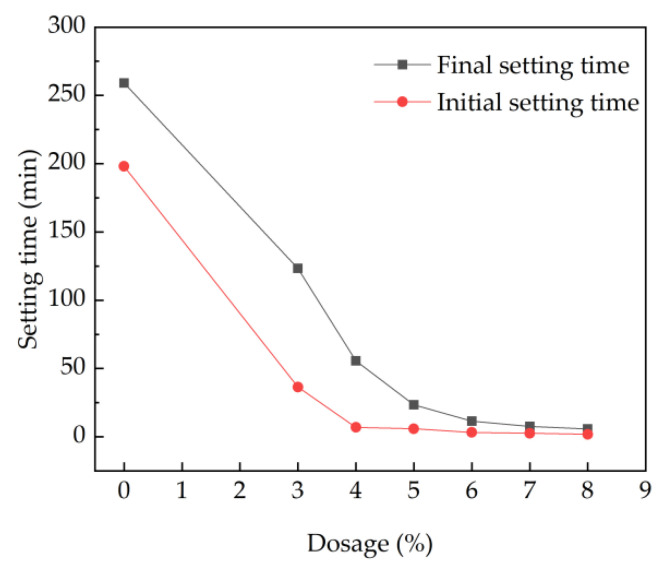
Effect of accelerator on the setting time of net cement paste.

The initial setting time and the final setting time of fresh cement paste are shown in Figure 2. As shown in Figure 2, the initial setting time and the final setting time decrease with the increasing dosages of the accelerator. As depicted in Figure 2, when the dosage of accelerating agent is 0%, the initial setting time is 198 min and the final setting time is 259 min. When the dosage is 3%, the setting time has an obvious downward trend, showing the initial and final setting time of 36.4 min and 123 min, with decreasing rates of 81.6% and 52.5%, respectively. The dosage of 4% is the threshold value. This is ascribed to the fact that when the dosage of accelerator is 3%, the polyaluminum sulfate is consumed in the hydration of cement, and the Ca^2+^ in cement minerals is not completely consumed. At the same time, the trisulfide hydrated calcium sulfoaluminate and hydrated calcium silicate generated cannot make the slurry set rapidly. Zhang et al. obtained a similar conclusion that the low dosage of accelerating agent resulted in less hydration production and the coagulation-promoting effect was not obvious [36,37].

**Figure 3:**
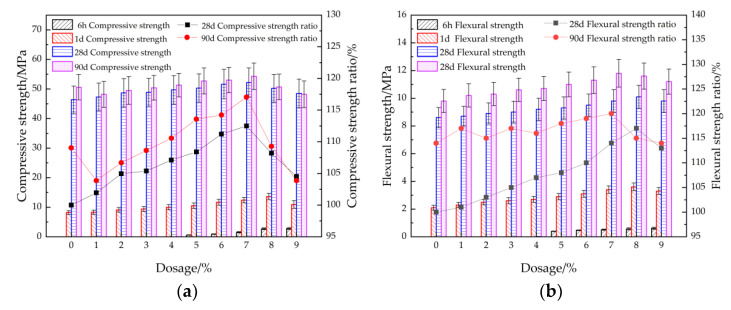
The mechanical strengths of cement mortar with accelerator. (**a**) Compressive strength, (**b**) flexural strength.

Figure 3 shows the compressive and flexural strengths of cement mortar with different dosages of accelerator with curing ages of 6 h, 1 d, 28 d and 90 d respectively. As observed from Figure 3, the compressive and flexural strengths of cement mortar increase with the increasing curing age and firstly increases and then decreases with the increasing addition of accelerator. When the dosage of accelerator is 7%, the mechanical strengths of the cement mortar is the highest. This is attributed to the fact that when the dosage of accelerator increases from 0% to 7%, a large amount of Al^3+^ and SO_4_^2−^ is increased by polyaluminum sulfate hydrolysis in the accelerator, which reacts quickly with Ca^2+^ produced by hydration of cement clinker and generates a large amount of needle-like hydrated 3CaO·Al_2_O_3_, forming a skeleton structure. Therefore, the mechanical strengths are increased. However, when the increasing dosages of accelerator is higher than 7%, the excessive SO_4_^2−^ induced by accelerator can react with Ca^2+^_,_ forming a large amount of dihydrate gypsum. The newly generated dihydrate gypsum can continue to react to generate a large amount of expansive hydrated calcium sulfoaluminate; therefore, the mechanical strengths are decreased by the addition of accelerator [38,39,40]. The error values are small and the test results are accurate.

### 3.2. Influence of Fly Ash on Basic and Compressive Performances

**Figure 4:**
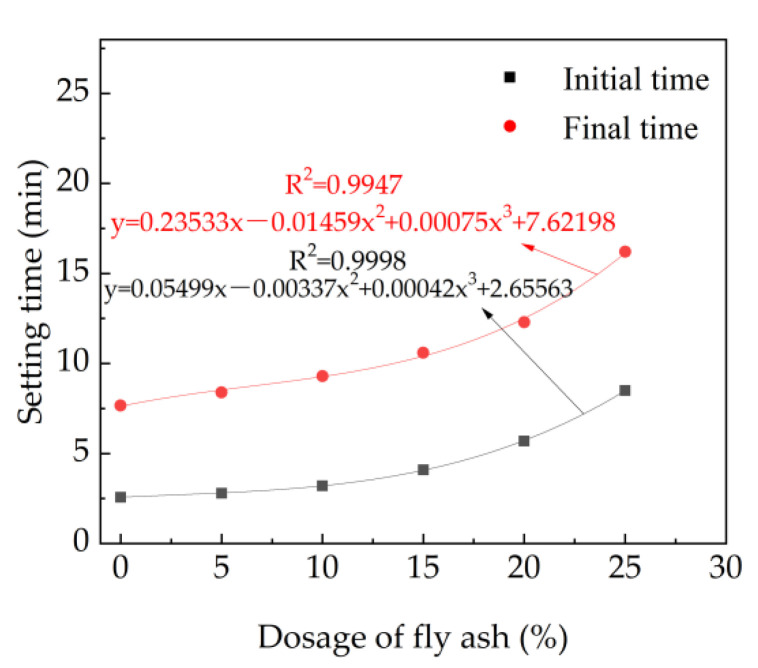
The setting time of fresh cement paste with fly ash.

The setting time of fresh cement paste with different dosage of fly ash and 7% accelerator is shown in Figure 4. As depicted in Figure 4, the initial and final setting time increase with the equation of the mass ratio of fly ash. This is attributed to the fact that the contents of 3CaO·SiO_2_, 2CaO·SiO_2_,3CaO·Al_2_O_3_ and 4CaO·Al_2_O_3_·Fe_2_O_3_ decrease with the addition of fly ash [41]. Moreover, as reported in prior research, fly ash contains active SiO_2_ and active Al_2_O_3_, and hydration reaction can occur only under the excitation of alkaline conditions, which makes the early activity of fly ash difficult to excite [42]. Therefore, the hydration rate of cement is decreased with the increasing dosages of fly ash, leading eventually to increasing the setting time, as observed in Figure 4, and relationships between the setting time and the mass rate of fly ash can be deduced as cubic functions.

**Figure 5:**
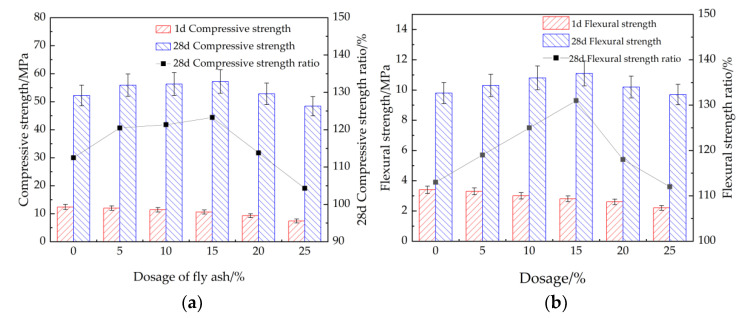
The mechanical strengths of cement mortar with fly ash. (**a**) Compressive strength, (**b**) flexural strength.

Figure 5 shows the compressive and flexural strengths of cement mortar with different dosages of fly ash. As illustrated in Figure 5, the compressive and flexural strengths of cement mortar cured for 1 day decrease with the increasing mass of fly ash. Meanwhile, when the curing age is 28 days, the compressive and flexural strengths of cement mortar firstly increase and then decrease with the increasing dosage of fly ash. Moreover, the pozzolanic activity of fly ash can lead to secondary hydration reaction, enhancing the late strength and making up for the loss of compressive strength of mortar [43]. The cement mortar with 15% fly ash shows the maximum compressive and flexural strengths. As illustrated in Figure 5, the values of error bars are lower than 0.09, indicating that the test results are accurate.

### 3.3. Influence of Blast Furnace Slag on Basic and Compressive Performances

**Figure 6:**
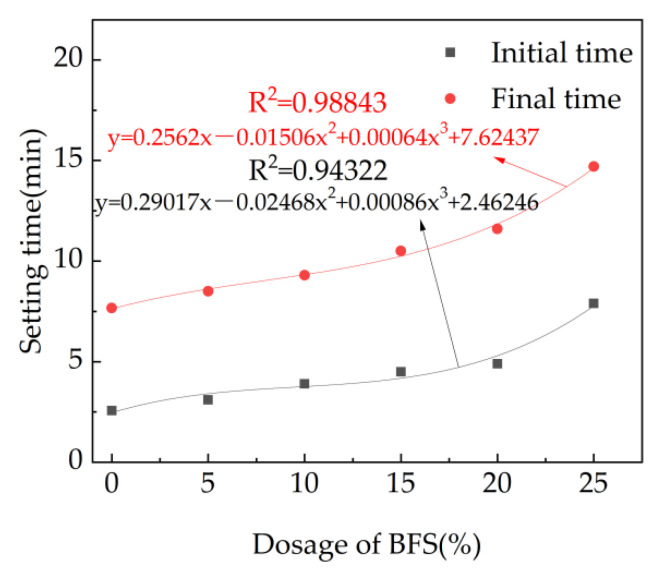
The setting time of fresh cement paste with BFS.

The initial setting time and final setting time of fresh cement paste with different dosages of blast furnace slag are shown in Figure 6. In this part, the accelerator is kept at 7% by mass of binder materials. It can be observed from Figure 6, the initial setting time and final setting time increase in the form of cubic function with the mass ratio of blast furnace slag, due to the decreased hydration rate by the addition of BFS, which is similar to the reason for fly ash.

**Figure 7:**
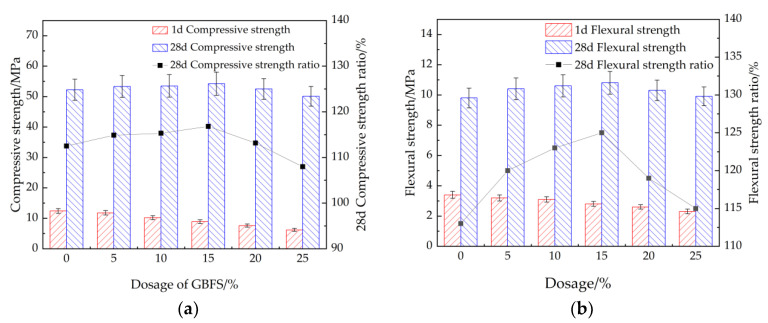
The mechanical strengths of cement mortar with BFS. (**a**) Compressive strength, (**b**) flexural strength.

Figure 7 shows the compressive and flexural strengths of cement mortar with 7% accelerator and different dosages of blast furnace slag. It can be observed from Figure 7 that the compressive and flexural strengths of cement mortar cured at 1 day decrease with the increasing dosage of BFS. However, when the curing age is 28 d, the compressive and flexural strengths firstly increase as the dosage of BFS increases from 0% to 15%. Meanwhile, when the dosage of BFS increases from 15% to 25%, the compressive and flexural strengths of cement mortar cured for 28 days decrease. Prior research points out that with the decreasing addition of slag, the amount of hydration products of early cement lead to reducing the early strength of cement. Moreover, with the increase of curing age, the active substances in the slag undergo secondary hydration in the alkaline environment, resulting in hydration products, which improves the compressive strength of the mortar in the later stage [44,45]. The error bars of 1 d compressive strength and flexural strength are lower than 0.8, which ensures the accuracy of the research results.

### 3.4. Heat of Hydration Analysis

**Figure 8:**
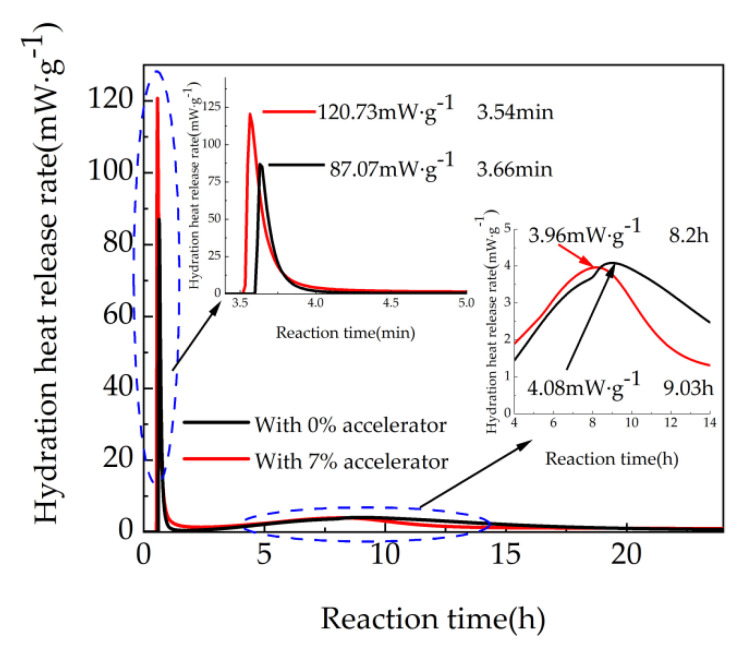
Hydration heat release rate curve.

According to the above results, the cement mortar with 7% accelerator shows the maximum mechanical strengths; therefore, in this part, cement mortar with 7% accelerator is selected for the measurement of heat of hydration. The hydration heat release rate curves are illustrated in Figure 8. It can be observed from Figure 8, the shapes of the hydration exothermic curves with 0% and 7% accelerator are similar. The first exothermic peak of the hydration exothermic curves with 0% and 7% accelerator appears in 3.66 min and 3.54 min, while the corresponding second exothermic peak appears in 9.03 h and 8.2 h. As shown in Figure 8, the first peak values of the two hydration exothermic curves are 120.73 m·Wg^−1^ and 80.07 m·Wg^−1^, respectively. Meanwhile, the second peak values of the two hydration exothermic curves are 3.96 m·Wg^−1^ and 4.08 m·Wg^−1^, respectively. The main reason is that Al^3+^ and SO_4_^2−^ in the accelerator consume Ca^2+^ in the liquid phase, leading to generating ettringite and releasing more heat and promoting the hydration of cement [44].

**Figure 9:**
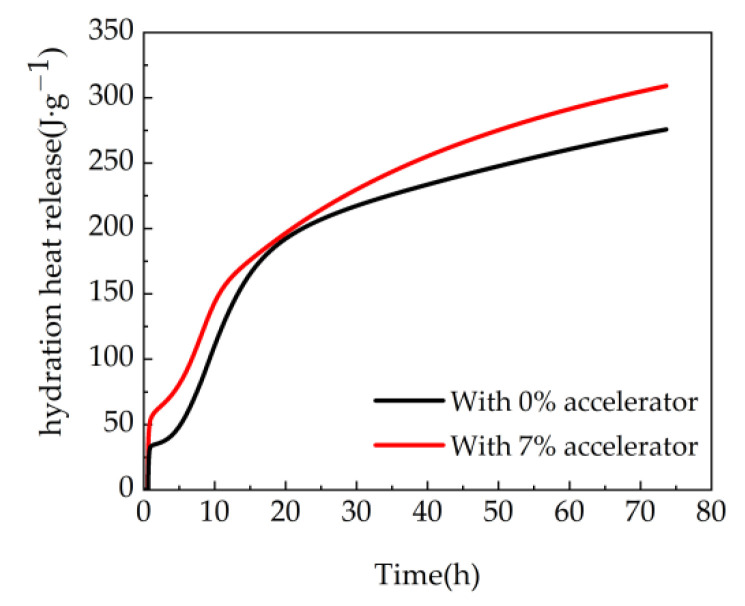
The hydration heat release of cement.

The total hydration heat release of cement is illustrated in Figure 9. It can be observed from Figure 9, the hydration heat release firstly increases sharply and then increases steadily with the time. This is attributed to the fact that the hydration rate of cement is relatively fast when at the early curing age. However, with the increasing curing age, the hydration rate can descend. The samples with 7% agent show higher hydration heat release than the blank samples. This is ascribed to the fact that the addition of accelerator leads to increasing the hydration reaction of 3CaO·Al_2_O_3_ in cement. Moreover, due to the pozzolanic activity of ultrafine nano-silica in the accelerator, the accelerator can react with Ca(OH)_2_ generating hydrated calcium silicate gel and improving the hydration rate [45]. Therefore, the total amount of hydration heat release of the experimental group is higher than that of the blank group.

### 3.5. XRD Pattern Analysis

**Figure 10:**
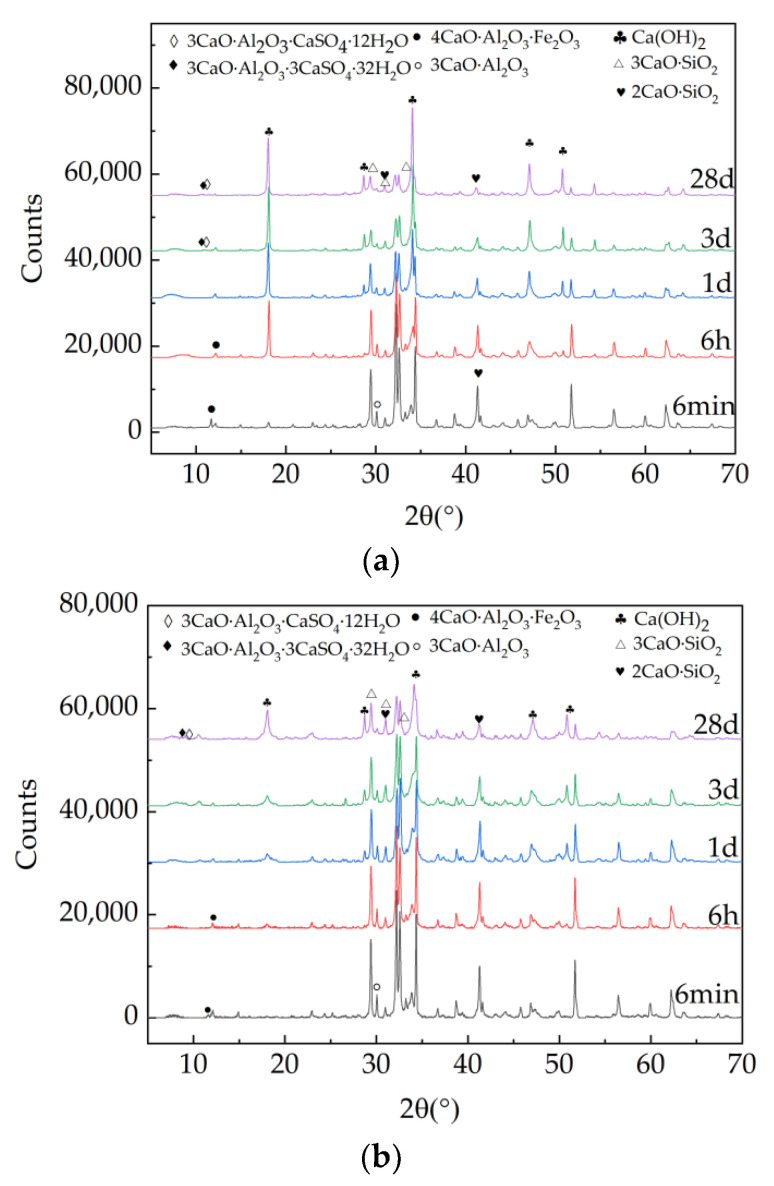
X-ray diffraction patterns of specimens. (**a**) X-ray diffraction pattern of blank group, (**b**) X-ray diffraction pattern of the sample with 7% accelerator.

Figure 10 shows the X-ray diffraction spectrum of blank cement paste and cement paste with 7% accelerator. It can be depicted in Figure 10, the 3CaO·SiO_2_, 2CaO·SiO_2_, 3CaO·Al_2_O_3_ and 4CaO·Al_2_O_3_·Fe_2_O_3_ can be found [46,47,48]. With the extension of curing age, the characteristic diffraction peaks of Ca(OH)_2_ became more obvious. Obvious 3CaO·Al_2_O_3_ diffraction peaks can be found in the samples with accelerator at all curing ages, indicating that a certain amount of 3CaO·Al_2_O_3_ is formed at 6 min of hydration. However, in the blank group, the aft diffraction peak could not be found until the curing age is 6 h.

**Figure 11:**
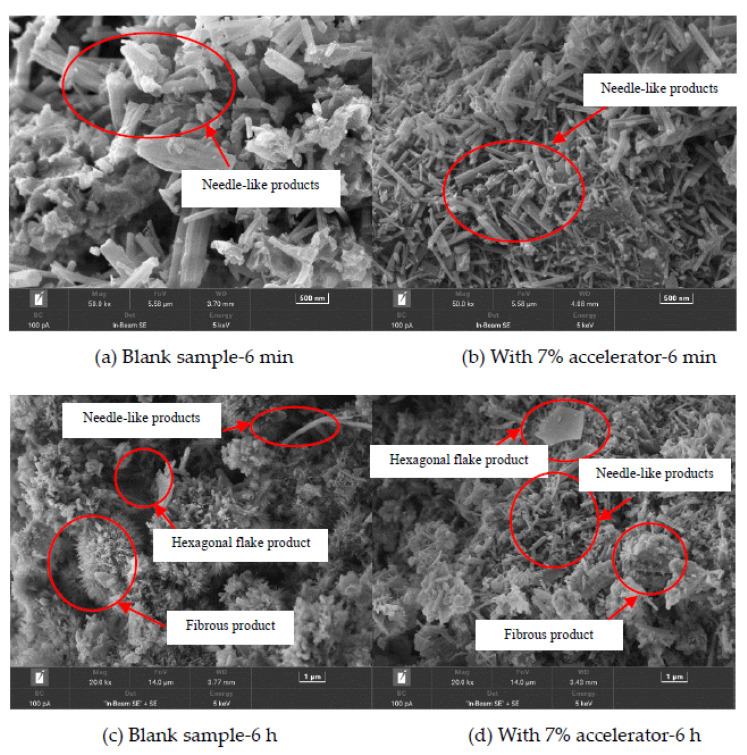

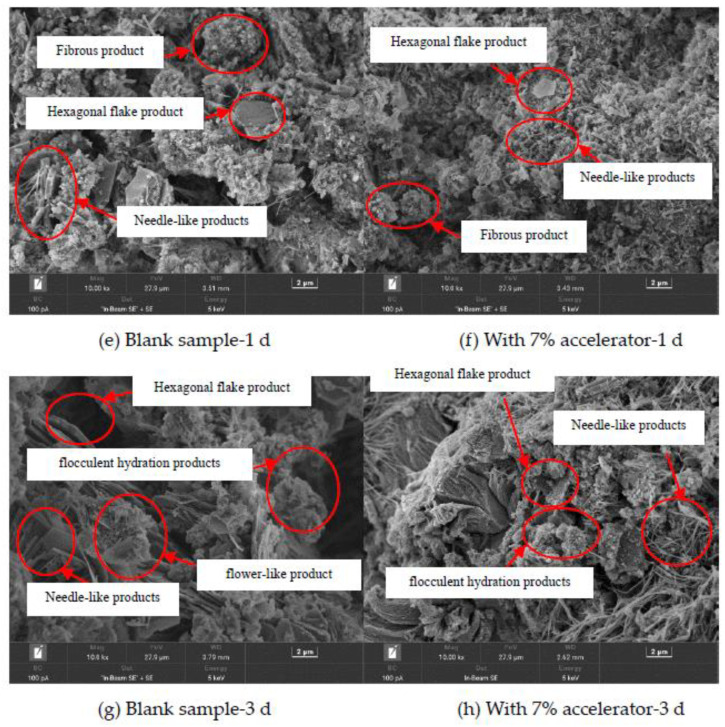

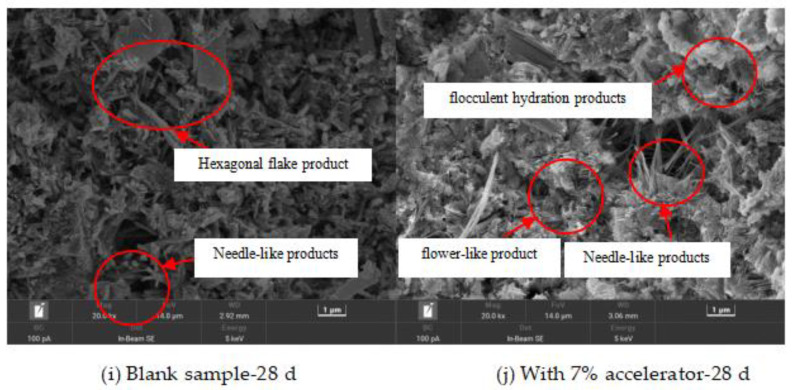
SEM images of specimens.

Figure 11 shows the scanning electron microscopy (SEM) photos of the blank specimens and the specimens with 7% accelerator cured for 6 min, 6 h, 1 d, 3 d and 28 d, respectively. It can be observed in Figure 11 that with increasing curing age, the amount of needle-like products decreases and the hexagonal flake and flocculent products increases, due to improved hydration degree. As depicted in Figure 11, the addition of accelerator leads to decreasing the needle-like products and improving the compactness of the hydration products at early curing age (the curing age is lower than 3 d). However, when the curing age is 28 d, little difference can be found in the SEM photos of the blank specimens and the specimens with 7% accelerator. This is because the accelerator can accelerate the early hydration process of the cement.

## 4. Conclusions

The purpose of this paper was to study the basic properties and micromechanisms of polyaluminum sulfate early strength alkali-free and fluorine-free liquid accelerator. The following conclusions are drawn.

The addition of accelerator can accelerate the setting of cement and improve the early strength of cement mortar. The initial setting time and the final setting time of cement paste are 2 min 30 s and 7 min 25 s. When the curing ages of cement mortar are 1 d, 28 d and 90 d, the compressive strengths of cement mortar are 2.4 MPa, 52.2 MPa and 54.3 MPa, and the corresponding flexural strengths are 3.4 MPa, 9.8 MPa, 11.8 MPa.

The setting time and mechanical strengths of cement past and cement mortar are affected obviously by the additions of accelerant, fly ash and blast furnace slag, where the dosages of accelerant, fly ash and blast furnace slag are 7%, 15% and 15%, respectively, and the mechanical strengths are the highest.

From the results of microscopic analysis, the addition of accelerating agent increases the hydration heat release rate of cement hydration and the overall hydration heat release in the hydration process. The accelerator can decrease the amount of needle like hydration products and improve the compactness. The mechanical strengths are improved by consuming a large amount of Ca(OH)_2_ and forming more compact hydration products.

This study has found a new type of early strength alkali-free liquid accelerator and the optimum dosages in the cement matrix. This technique will be applied in actual projects in the future. These will provide technical support and theoretical basis for the preparation and application of alkali-free liquid accelerators in the future.

## Figures and Tables

**Table 1:** Chemical compositions of cement (%).

Materials	SiO_2_	Al_2_O_3_	Fe_2_O_3_	CaO	MgO	SO_3_	Na_2_O_qe_	f-CaO
Cement	21.6	4.6	3.9	63.7	3.2	2.3	0.52	0.95
Fly ash	51.24	34.12	4.23	2.9	0.62	1.21	1.83	0.54
BFS	33.21	17.32	0.84	36.21	9.34	2.82	0.89	0.019

**Table 2:** The mix of specimens per unit volume.

Cement	Fly Ash	Slag	Standard Sand	Water	Accelerated Agent
100	0	0	0	33.5	3
100	0	0	0	33	4
100	0	0	0	32.5	5
100	0	0	0	32	6
100	0	0	0	31.5	7
100	0	0	0	31	8
95	5	0	0	31.5	7
90	10	0	0	31.5	7
85	15	0	0	31.5	7
80	20	0	0	31.5	7
75	25	0	0	31.5	7
95	0	5	0	31.5	7
90	0	10	0	31.5	7
85	0	15	0	31.5	7
80	0	20	0	31.5	7
75	0	25	0	31.5	7
100	0	0	150	50	0
100	0	0	150	49.5	1
100	0	0	150	49	2
100	0	0	150	48.5	3
100	0	0	150	48	4
100	0	0	150	47.5	5
100	0	0	150	47	6
100	0	0	150	46.5	7
100	0	0	150	46	8
100	0	0	150	45.5	9
95	5	0	150	46.5	7
90	10	0	150	46.5	7
85	15	0	150	46.5	7
80	20	0	150	46.5	7
75	25	0	150	46.5	7
95	0	5	150	46.5	7
90	0	10	150	46.5	7
85	0	15	150	46.5	7
80	0	20	150	46.5	7
75	0	25	150	46.5	7

## Data Availability

The data used to support the findings of this study are available from the corresponding author upon request.
